# Long-Term Incidence of Venous Thromboembolism in Cancer: The Scandinavian Thrombosis and Cancer Cohort

**DOI:** 10.1055/s-0038-1641678

**Published:** 2018-04-06

**Authors:** Inger Lise Gade, Sigrid K. Brækkan, Inger Anne Næss, John-Bjarne Hansen, Suzanne C. Cannegieter, Frits R. Rosendaal, Kim Overvad, Kristian Hindberg, Jens Hammerstrøm, Olga V. Gran, Anne Tjønneland, Marianne T. Severinsen, Søren R. Kristensen

**Affiliations:** 1 Department of Clinical Medicine, Aalborg University, Aalborg, Denmark; 2 Department of Clinical Medicine, K.G Jebsen Thrombosis Research and Expertise Centre (TREC), University of Tromsø—The Arctic University of Norway, Tromsø, Norway; 3 Division of Internal Medicine, University Hospital of North Norway, Tromsø, Norway; 4 Department of Haematology, Trondheim University Hospital, Trondheim, Norway; 5 Department of Clinical Epidemiology, Leiden University Medical Center, Leiden, The Netherlands; 6 Section for Epidemiology, Department of Public Health, Aarhus University, Aarhus, Denmark; 7 Department of Cardiology, Aalborg University Hospital, Aalborg, Denmark; 8 Department of Cancer Research and Molecular Medicine, Norwegian University of Science and Technology, Trondheim, Norway; 9 Department of Diet, Genes and Environment, Danish Cancer Society Research Center, Copenhagen, Denmark; 10 Department of Haematology, Aalborg University Hospital, Aalborg, Denmark; 11 Department of Clinical Biochemistry, Aalborg University Hospital, Aalborg, Denmark

**Keywords:** cancer, epidemiology, prospective studies, time factors, venous thromboembolism

## Abstract

The risk of venous thromboembolism (VTE) in patients who survive the first years after a cancer diagnosis after the acute effects of disease and treatment in comparison to a similar background population has been sparsely investigated. The aim of the study was to investigate if incidence rates (IRs) of VTE differed in patients who were alive at least 2 years after a cancer diagnosis without VTE compared with cancer-free references in a population-based cohort study. The study entry was 2 years after a first cancer diagnosis. For each cancer-exposed subject, five reference subjects were identified within the cohort. The IRs were calculated as number of VTEs per 1,000 person years (×10 ^−3^ p-y) in total and in distinct cancer types and corresponding reference subjects. Incidence rate ratios (IRRs) were calculated by Poisson's regression. During a mean follow-up of 5.3 years, 110 VTEs occurred among the 7,288 cancer-exposed subjects and 321 VTEs occurred among the 36,297 identified reference subjects. The IR of VTE was higher for cancer-exposed subjects compared with reference subjects, IRs 3.7 × 10 ^−3^ p-y, 95% CI: 3.1 to 4.5 and 1.9 × 10 ^−3^ p-y, 95% CI: 1.7 to 2.2, respectively. IRs of VTE in most solid cancer types declined to almost the same level as in the reference subjects 2 years after cancer diagnosis, but remained higher in hematological cancers, IRR 4.0, 95% CI: 2.0 to 7.8.

## Introduction

Cancer entails a higher risk of venous thromboembolism (VTE) and the risk varies due to patient-specific, cancer-specific, and treatment-related risk factors, and depends on time since cancer diagnosis. 1 2 3 4 5 6 7 8 The risk of VTE is highest in the months shortly before and after cancer diagnosis and attenuates considerably by time since cancer diagnosis. 2 7 9 10 11 However, the incidence of VTE among cancer patients who survive the first years after a cancer diagnosis in comparison to the background population is sparse.

The risk of VTE in association with a cancer diagnosis is strongly related to cancer type, and consequently the risk of VTE in patients who are alive when the acute effect of the cancer and its treatments on the risk of VTE attenuates may also differ according to cancer type. Several studies on single entities of cancer have reported risk of VTE associated with cancer and typically the occurrence of VTE was described as the cumulative incidence proportions of VTE with reference to the time since cancer diagnosis. 10 11 12 13 14 15 16 17 18 19 20 21 This measure is, however, not suitable for the estimation of the VTE risk for cancer patients who pay their last visit in the oncologic outpatient clinic after relapse-free survival some years after the diagnosis. If the VTEs in close proximity to the cancer diagnosis are included, this estimate will be too high. For patients who are alive after the acute effect of cancer and the associated treatments have faded, the early events in the cancer group should not be included when estimating their future risk of VTE.

The Scandinavian Cancer and Thrombosis (STAC) cohort contains person-time data for 144 952 persons among whom 19,757 cancers occurred during a median follow-up of 14.1 years. 22 The size of the STAC cohort enables matching to cancer-free subjects within the cohort and thus comparison of VTE incidence rates (IRs) in subjects with different initial cancer types.

The objective of this study was to investigate the incidence of VTE in cancer-exposed subjects who survived the first 2 to 5 years after cancer diagnosis without VTE. These patients contributed to person–time at risk of first lifetime VTE from 2 years after cancer diagnosis and onwards. IRs of VTE were estimated for the cancer-exposed subjects overall and in distinct types of cancer and were compared with the IRs of VTE in cancer-free reference subjects starting at different time points from initial cancer diagnosis.

## Methods

### Study Population

Prospectively collected data from three large population-based studies were merged in the STAC cohort. The Danish Diet, Cancer and Health (DCH) Study contributed with 57,053 participants; the Norwegian studies, the Tromsø Study (Tromsø 4), and the Nord-Trøndelag Health Study (HUNT2) contributed with 27,158 and 65,237 participants, respectively. In the two Norwegian cohorts, all inhabitants of Tromsø and Nord-Trøndelag between 25 to 99 and 19 to 103 years of age, respectively, were invited to participate, while in the DCH study all inhabitants of the cities Copenhagen and Aarhus aged 50 to 64 were invited. The rates of participation were between 35% and 77%, highest in Tromsø. 23 24 25 Enrolment took place from 1993 through 1997, and subjects with prior cancer or VTE were excluded from the STAC cohort, leaving 144,952 participants to follow up. By informed, written consent at enrolment, every participant permitted linkage to national registries by the civil personal registration (CPR) number, which is a unique id assigned to every citizen at birth or immigration by the respective national civil registration systems. They are continuously updated on vital and emigration status, which means no de facto loss to follow-up in studies taking advantage of the CPR numbers. Recently, the STAC cohort's profile including age-specific IRs of cancer and VTE was published. 22 The age-standardized rates of cancer in the three original cohorts did not differ from the respective general populations. In this study, we included all cancer patients, who were alive and free of previous VTE 2 years after a first cancer diagnosis. Patients in the STAC cohort with a cancer diagnosis after last follow-up for validated VTEs ( *n* = 301) were excluded, leaving in a total of 7,645 patients with a former cancer diagnosis eligible for this study.

### Identification of Cancer-Exposed Subjects and Sampling of Reference Subjects

In both Denmark and Norway, cancer registration is mandatory by law and the respective national cancer registries provide high-quality data on individual cancer diagnosis date and cancer type by ICD-10 codes, which identified cancer patients in this study. 26 27 28 29 30 All cancer types were included except nonmelanoma skin cancers (ICD-10 C 44.x) and chronic myeloproliferative/myelodysplastic diseases (ICD-10 C 92.1–C 45.9 and D 45.0–47.3) as this group was not registered in the national cancer registries before 2004. If a new cancer was registered by an ICD-10 code different from the index cancer after the study entry, this was considered a second cancer. Cancer stage was registered by national classification systems, the Federation of Gynecology and Obstetrics (FIGO) classification (for gynecological tumors), Duke's classification (for colorectal cancer), or the Tumor, Node, Metastasis (TNM) classification. We have recently developed an algorithm for mapping of cancer stages in the different classification systems to a “localized, regional spread, distant metastasis” terminology according to recommendations from the International Cancer Benchmark Partnership. 31 32

For each cancer-exposed subject, we intended to identify five reference subjects in the STAC cohort free of previous cancer and VTE at the index date. Reference subjects were matched on age at study entry, sex, and original study to control for confounding by these factors, the latter due to different points of administrative censoring in the three original cohorts. In case no reference subjects were identified, the cancer-exposed subject was excluded from the study.

### Outcome

All potential first-time, symptomatic VTE events (pulmonary embolism and deep vein thrombosis covered) in the STAC cohort were identified before merging by linkage of CPR numbers to hospital discharge registries and radiology procedure registries in Norway and the Danish National Patient registry and the Danish national Cause of Death Registry in Denmark. Subsequently all VTE events were validated by review of medical records; typical symptoms of VTE, biochemical tests and diagnostic images, and provoking factors were noted. A VTE was confirmed in cases where a diagnostic test following typical clinical symptoms confirmed a VTE as described in detailed in previous publications. 33 34 35 Last follow-up for VTE was in 2012 in the Tromsø Study, in 2008 in DCH, and in 2007 in HUNT2.

### Statistics

The study entry was set to 730.50 days after first cancer diagnosis and the corresponding index date for reference subjects. Participants contributed with cancer–exposed and corresponding never-cancer–exposed person-time until VTE, end of follow-up for VTE, second cancer diagnosis, emigration or death, and for reference subjects a first cancer diagnosis, whichever came first. We calculated IRs as number of VTEs per 1,000 person-years (×10 ^−3^ p-y) for different time periods after cancer diagnosis: the entire study period (i.e., ≥ 2 years after cancer diagnosis) and 3 years after study entry (i.e., ≥5 years after cancer diagnosis) and at corresponding points in reference subjects. Incidence rate differences (IRD) and associated 95% confidence intervals (CI) were calculated to describe absolute differences in VTE occurrence in cancer-exposed subjects and reference subjects. Estimates were calculated for all cancers combined and for the most common single entities of cancer among the cancer-exposed subjects: hematological cancers, colorectal, breast, and prostate cancers. All other solid cancer types were combined in one group with lung, bladder, and uterine cancers being the most common cancer types.

Relative measures of association were calculated as incidence rate ratios (IRR) with associated 95% CIs. Age was included as a continuous variable represented as a restricted cubic spline variable with four knots in the Poisson regression model to control for aging since study entry/index date. The association between initial cancer stage (at the time of diagnosis) and VTEs in the cancer-exposed subjects was assessed in separate analysis.

To illustrate the absolute risk of VTE, cumulative incidence proportions of VTE since study entry were calculated for reference subjects and for cancer overall. We created variables containing the cumulative incidences of each of the censoring events; death and second cancer were treated as competing risks in the cancer exposed, while death and first cancer were treated as competing risks in reference subjects.

The significance of the difference between provoked VTEs in cancer-exposed subjects compared with reference subjects was tested by using chi-square test.

## Results

Mean age for the cancer-exposed subjects at study entry (i.e., 2 years after cancer diagnosis) was 68.9 years, and 49.5% were males. The vast majority of the cancer-exposed subjects had initially (i.e., at the time of diagnosis) localized or regional spread cancer ( Table 1 ). During the study period, a cancer diagnosis occurred in 4,855 (13.4%) of the reference subjects. A second cancer diagnosis occurred in 770 (10.6%) of the cancer-exposed subjects during the study period and three VTEs occurred after this diagnosis. In total 431 VTEs occurred during the study period with a mean follow-up of 5.3 years (i.e., 7.3 years after cancer diagnosis). A total of 110 of the VTEs occurred among the cancer-exposed subjects; 77 of these occurred more than 1 year after study entry (i.e., more than 3 years after cancer diagnosis) and 44 occurred later than 3 years after study entry (i.e., more than 5 years after cancer diagnosis).

**Table 1:** Baseline characteristics for the cancer-exposed subjects and the reference subjects

	*N*	Male	Female	Mean age, y (range)	SD mean age	Localized disease % ( *n* )	Regional spread % ( *n* )	Distant metastasis % ( *n* )	Unknown stage % ( *n* )
Reference subjects	36,297	16,506	19,791	65.0 (22.0–97.0)	9.5	–	–	–	–
Breast	1,703	9	1,694	63.5 (35.5–97.1)	8.9	51 (872)	36 (621)	2 (28)	11 (182)
Prostate	1,331	1,331	0	69.0 (44.5–97.4)	6.9	49 (658)	10 (130)	10 (127)	31 (416)
Colorectal	1,122	561	561	67.8 (38.0–93.1)	8.9	39 (442)	50 (559)	5 (57)	6 (64)
Other solid cancer	2,571	1,122	1,449	64.3 (23.9–94.9)	10.7	64 (1,636)	16 (420)	7 (179)	13 (336)
Hematological	561	295	266	65.7 (22.5–96.5)	11.3	na	na	na	na

Abbreviations: na, not available; SD, standard deviation.

In total, 36,297 reference subjects were identified. For 7,238 of the 7,645 identified cancer-exposed subjects, five reference subjects were identified, but despite the size of the STAC cohort, no reference subjects were available for 357 cancer-exposed subjects which were excluded from the study. The majority of these were older males from the Tromsø Study. For 22 cancer exposed, one reference subject was identified, for 9 cancer exposed two reference subjects, for another 9 cancer exposed three reference subjects, and for 10 cancer exposed four reference subjects were identified.

**Fig. 1:**
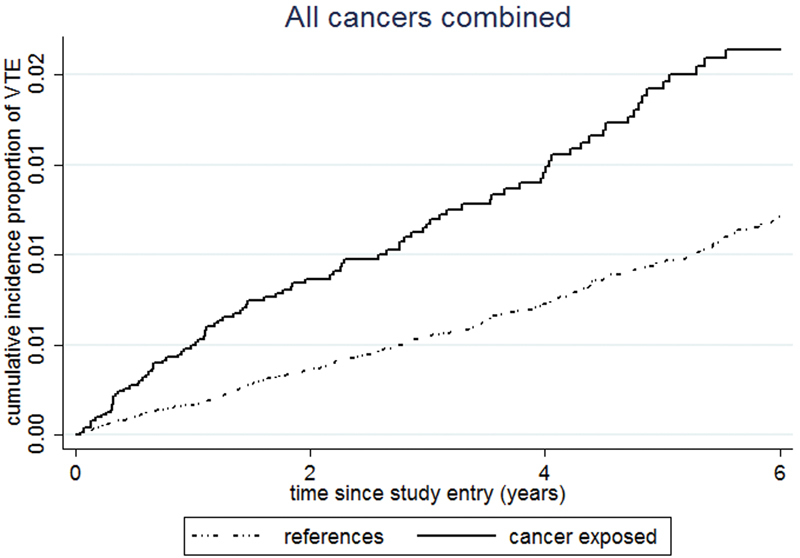
Cumulative incidence proportion of venous thromboembolism (VTE) in cancer-exposed subjects and reference subjects. Death as well as first and second cancer, respectively, is treated as competing risks in reference subjects and cancer survivors.

The IR of VTE that occurred during the total study period (i.e., from more than 2 years after cancer diagnosis) was higher in the cancer-exposed subjects (IR: 3.7 × 10 ^−3^ p-y, 95% CI: 3.1–4.5) compared with the reference subjects (IR: 1.9 × 10 ^−3^ p-y, 95% CI: 1.7–2.2; Table 2 ). The cumulative incidence proportion of VTE increased linearly during follow-up for both cancer-exposed and reference subjects, but with a steeper slope in cancer exposed ( Fig. 1 ). The IRs of VTEs calculated for the periods ≥ 2 and ≥5 years after a cancer diagnosis were all nearly twofold higher compared with reference subjects ( Table 3 ). The absolute occurrence of VTE was significantly higher in cancer-exposed compared with reference subjects even when restricting observation time to the period beyond 5 years after cancer diagnosis (IRD: 1.3 × 10 ^−3^ p-y, 95% CI: 0.2–2.3).

**Table 2:** IRs and IRDs of VTE in cancer-exposed subjects and their matched reference subjects by increasing time since cancer diagnosis

	Total study period, i.e., ≥ 2 y after cancer diagnosis				≥ 5 y after cancer diagnosis	
	Total VTE, *n*	Person time, y	IR per 1,000 p-y (95% CI)	IRD (95% CI)	IR per 1,000 p-y (95% CI)	IRD (95% CI)
**All reference subjects combined**	**321**	**165,180**	**1.9 (1.7–2.2)**	**Ref.**	**2.1 (1.8–2.4)**	**Ref.**
All cancer-exposed combined	110	30,021	3.7 (3.1 **–** 4.5)	1.8 (1.1 to 2.5)	3.3 (2.5 **–** 4.5)	1.3 (0.2 to 2.3)
Breast	19	8,098	2.3 (1.5 **–** 3.7)	0.9 (− 0.3 to 2.0)	2.0 (1.0 **–** 4.1)	1.0 (–0.5 to 2.5)
Prostate	18	4,619	3.9 (2.5 **–** 6.2)	1.4 (− 0.5 to 3.3)	3.3 (1.5 **–** 7.4)	0.3 (–2.6 to 3.2)
Colorectal	21	4,294	4.9 (3.2 **–** 7.5)	2.6 (0.5 to 4.8)	2.6 (1.1 **–** 6.4)	–0.6 (–3.1 to 1.9)
Other solid tumors	40	10,506	3.8 (2.8 **–** 5.2)	1.8 (0.6 to 3.1)	3.9 (2.5 **–** 6.2)	1.8 (0.0 to 3.7)
Hematological	12	1,853	6.5 (3.7 **–** 11.4)	4.9 (1.2 to 8.7)	8.0 (3.6 **–** 17.8)	6.3 (–0.3 to 12.7)

Abbreviations: CI, confidence interval; IR, incidence rate; IRD, incidence rate differences; p-y, person-year; Ref., reference; VTE, venous thromboembolism.

**Table 3:** Crude and adjusted IRRs of VTE in cancer-exposed subjects by increasing time since cancer diagnosis

	Total study period, i.e., > 2 y after cancer diagnosis				> 5 y after cancer diagnosis			
	IRR, crude	95% CI	IRR, adjusted a	95% CI	IRR, crude	95% CI	IRR, adjusted a	95% CI
**All reference subjects combined**	**Ref.**	**–**	**Ref.**	**–**	**Ref.**	**–**	**Ref.**	**–**
All cancer-exposed combined	1.8	1.5–2.2	1.8	1.5–2.2	1.8	1.4–2.4	1.8	1.4–2.4
Breast	1.7	1.1–2.7	1.7	1.0–2.6	2.6	1.3–5.0	2.5	1.3–4.9
Prostate	1.4	0.9–2.3	1.4	0.9–2.3	1.4	0.7–2.9	1.5	0.7–2.9
Colorectal	1.7	1.1–2.7	1.7	1.0–2.6	0.8	0.3–1.8	0.8	0.3–1.8
Other solid cancer	2.1	1.3–3.3	2.1	1.3–3.3	2.1	1.3–3.3	2.1	1.3–3.3
Hematological	4.0	2.0–7.9	4.2	2.2–8.1	4.7	1.9–11.6	5.2	2.1–12.4

Abbreviations: CI, confidence interval; IRR, incidence rate ratios; Ref., reference; VTE, venous thromboembolism.

a Adjusted for age since study entry.

In the single entities of cancer, we observed varying IRs of VTE in the cancer-exposed subjects, highest in the hematological cancers (IR VTE ≥ 2 years after cancer diagnosis 6.5 × 10 ^−3^ p-y, 95% CI: 3.7–11.4). The corresponding IRRs of VTE in hematological cancers compared with their reference subjects in the total study period were also high (IRR: 4.0, 95% CI: 2.0–7.9) ( Table 3 ). Thus, the steeper slope of the cumulative incidence proportion of VTE in cancer-exposed subjects compared with reference subjects was mainly driven by the VTEs in hematological cancers. The IRs of VTE in subjects exposed to solid tumors were in general lower than in hematological cancers, but remained higher than reference subjects in colorectal and the group of other solid cancer types when VTEs that occurred at least 2 years after cancer diagnosis were included ( Table 2 ). The IRs of VTE in prostate and colorectal cancer resembled that observed in the reference subjects by successive time since cancer diagnosis ( Table 2 ). We observed no residual confounding by the aging since cancer diagnosis/index date ( Table 3 ).

The impact of initial cancer stage at diagnosis on the IR of VTEs in subjects exposed to solid cancer types was assessed in a separate analysis. The IRRs of VTE in subjects exposed to regional spread and distant metastatic cancers were 2.4 (95% CI: 1.7–3.5) and 5.1 (95% CI: 2.7–9.5), respectively, compared with their reference subjects for the period 2 years after cancer diagnosis, which was higher than the subjects exposed to localized disease (1.5, 95% CI: 1.1–2.0). The IRRs for subjects exposed to initial regional spread or distant metastatic cancer tended to be lower >5 years after cancer diagnosis ( Table 4 ).

**Table 4:** Crude and adjusted IRRs of VTE stratified on initial cancer stage in cancer-exposed subjects compared with their controls by increasing time since cancer diagnosis

	Total study period, i.e., > 2 y after cancer diagnosis					> 5 y after cancer diagnosis			
	VTE ( *n* )	IRR, crude	95% CI	IRR, adjusted a	95% CI	IRR, crude	95% CI	IRR, adjusted a	95% CI
Localized disease	43	1.5	1.1–2.0	1.2	0.6–2.2	2.0	1.3–2.9	2.0	1.0–4.2
Regional spread	36	2.4	1.7–3.5	1.9	1.0–3.6	1.5	0.8–2.7	1.4	0.5–3.9
Distant metastasis	13	5.1	2.7–9.5	6.3	1.4–28.5	3.7	1.2–11.3	3.3	1.0–10.8
Actively coded “Unknown stage”	18	1.4	0.9–2.2	0.8	0.2–3.2	1.8	1.0–3.3	0.8	0.1–5.6

Abbreviations: CI, confidence interval; IRR, incidence rate ratios; Ref., reference; VTE, venous thromboembolism.

a Adjusted for age since study entry and cancer type.

In the cancer-exposed subjects, 39.1% of the VTEs were provoked as compared with 36.4% in the reference subjects. This small excess of provoked VTE was not statistically significant ( *p* = 0.62).

## Discussion

In this population-based cohort, IRs of VTE in cancer-exposed subjects alive 2 years after their cancer diagnosis and free of VTE were compared with age- and gender-matched reference subjects. The IR of VTE was higher in all cancer-exposed subjects combined compared with the reference subjects in both study periods. However, in the solid cancer types, the IR fell successively by increasing time after cancer diagnosis resembling the IRs of VTE of their reference subjects over time, whereas in hematological cancers the IR of VTE remained higher in cancer-exposed subjects compared with reference subjects throughout the study period. Initial distant metastasis was associated with a fivefold higher risk of developing a first time VTE more than 2 years after cancer diagnosis.

The hematological cancers encompassed a large proportion of multiple myeloma and chronic lymphocytic leukemia, which are incurable diseases repeatedly treated at relapses. Myeloma is well known for the associated high risk of VTE, and recent studies have shown that chronic lymphocytic leukemia may also be associated with a high incidence of VTE. 4 36 37 The high long-term incidences of VTE in the hematological cancers might be due to anticancer treatments, but since IMID-based combination therapy typically did not persist for years in the study period and guidelines recommended thromboprophylaxis, other factors may also contribute. It has been suggested that cancer cells contribute to hypercoagulability by releasing procoagulant microvesicles. 38 This may be associated with disease activity, and the activity fluctuates in the incurable hematological cancers. Therefore, this may explain a persistently increased risk of VTE several years after cancer diagnosis in the group of hematological cancers. Along the same line, the observed persistently higher risk of VTE in subjects exposed to initial metastatic cancer is probably, still to some degree, attributable to cancer activity or treatments.

It is relevant to appreciate the validated outcomes in our study since a validation study has shown that discharge diagnoses of VTE from the National Patient Registry had a positive predictive value of approximately 75%. 35 Consequently direct comparison of IRs of VTE in this study with other studies is difficult. A recent population-based Swedish study on time-dependent risk of VTE in breast cancer patients found a 5-year cumulative incidence of 4.0% in breast cancer patients, while in age-matched controls the 5-year cumulative incidence of VTE was 1.1%. The presented measures of association were calculated from date of cancer diagnosis, thus encompassing the large proportion of VTEs that occurred during the first year of the study period. 14 As we started our study 2 years after cancer diagnosis and further analyzed at points corresponding to 5 years after cancer diagnosis, the former VTEs did not contribute to the association measures in our study, and consequently the IRs of VTE were only marginally higher in breast cancer–exposed subjects compared with their reference subjects. In the Swedish study, the cumulative incidence curves of VTE that occurred later than 2 years after breast cancer/index date is only slightly steeper for the breast cancer exposed than for the matched cohort reflecting marginally increased risk of VTE in breast cancer–exposed subjects, in accordance with our results. In a Danish population-based cohort study, the reported IRs of VTE that occurred more than 2 years after cancer diagnosis were in general higher than we observed. 3 Their use of discharge diagnoses from the National Patient Registry, a broader definition of VTE and no censoring in case of a second primary cancer diagnosis, may explain this, although the variation is minor. A recent Danish population-based study on VTE in prostate cancer found that a low to moderate comorbidity in combination with cancer could increase the risk of VTE more than expected from their individual effects. 39 The reported rates and rate ratios of VTE were similar to our results. However, we did not have the opportunity to include comorbidity in our study, which would have been beneficial.

The results from our study should be interpreted within its limitations, of which the lack of information on comorbidity, anticoagulation, and chemotherapy are the most important. Furthermore, we have too few events in some groups to give meaningful estimates of IR and associated association measures, which is also the reason why hematological cancers were combined in one group despite their varying phenotypes and risks of VTE. The myeloproliferative and myelodysplastic disorders were not included because they were not available in the registries, but we would expect the number of VTEs to be very limited in these groups. We cannot rule out misclassification due to second primary cancers, but this would probably be nondifferential and only comprise a minor proportion which most probably would not affect the presented estimates of occurrence and association. Another limitation is the inability to obtain valid information on cancer relapses from the national cancer registries and we may, therefore, overestimate the IRs of VTE in the cancer-exposed subjects. This would expectedly, in particular, be an important factor among those exposed to hematological cancer types. On the other hand, despite the potential overestimation of IR of VTE in the cancer-exposed subjects, the IRs of VTE in subjects exposed to breast, colorectal, and prostate cancer were not significantly higher compared with their reference subjects 5 years after the cancer diagnosis.

We know from unpublished data that 127 of VTEs observed in the STAC cohort occurred within 1 year before a first cancer diagnosis. Seventeen of these VTEs counted as events among reference subjects in this study; when compared with the total number of 321 VTEs in the reference subjects, they will have a minor effect on the resulting increased IR of VTE in the reference group and would draw our results toward the null hypothesis. The cancer-exposed group is a mix of cured and uncured patients, and the risk of VTE years after a cancer diagnosis would expectedly differ by this fact as reflected in the different risks of VTE according to cancer types and initial cancer stage. The proportion of cured and uncured patients in our cohort should, however, resemble the mix of cancer patients usually seen in hospitals.

Successively improved cancer treatments and accelerated cancer-diagnosing programs have increased the proportion of 5-year cancer survivors steadily during the past years. 40 With these recent advances, it is becoming increasingly relevant to describe health among cancer survivors. Our observations are particularly relevant for the cancer patients having survived to be discharged from the oncologic outpatient clinics, which would be at 3 to 5 years for most cancer types (in the Danish health care system).

To the best of our knowledge, this is the first population-based study to specifically investigate the incidence of VTE in subjects exposed to cancer being alive when the acute effect of the cancer and its treatments on the risk of VTE attenuated some years after the diagnosis. In conclusion, our study indicates that the risk of a first VTE in cancer-exposed subjects alive more than 2 years after their cancer was diagnosed is modestly elevated compared with the background population, a finding mainly due to sustained VTE occurrence in the hematological cancers.
